# Supplementary figures and images for: Single-nucleus RNA-seq identifies Huntington disease astrocyte states
Source: Acta Neuropathol Commun. 2020 Feb 18;8:19. doi: 10.1186/s40478-020-0880-6 (PMC7029580; doi:10.1186/s40478-020-0880-6)

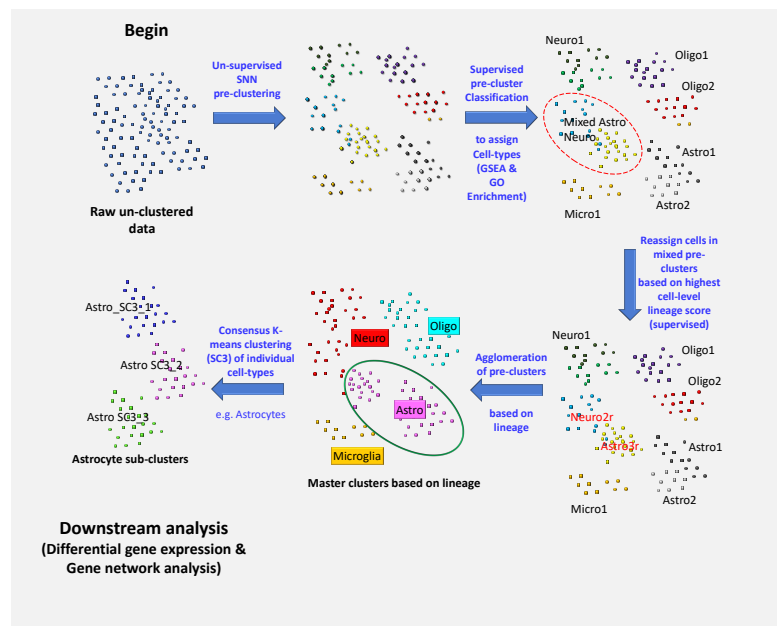

Supplement: Supplementary file 1 — Additional file 1. Outline of the snRNAseq analysis pipeline. Briefly, filtered raw un-clustered data is pre-clustered using a shared nearest neighbor algorithm. The pre-clusters are classified into cell classes/lineages using gene set enrichment analysis for specific lineage genes and examining GO terms of the top pre-clusters markers. Next, mixed pre-clusters are identified and the cells in these pre-clustered are re-classified based on the cell-specific lineage-scores (Cell classifier tool). Next, clusters of the same lineage are agglomerated and cell-classes/lineages are analyzed in isolation from the remaining cell classes using SC3 consensus clustering into sub-clusters (Astrocyte sub-clusters are shown as an example). These sub-clusters are used for downstream analysis. [file 40478_2020_880_MOESM1_ESM.pdf]

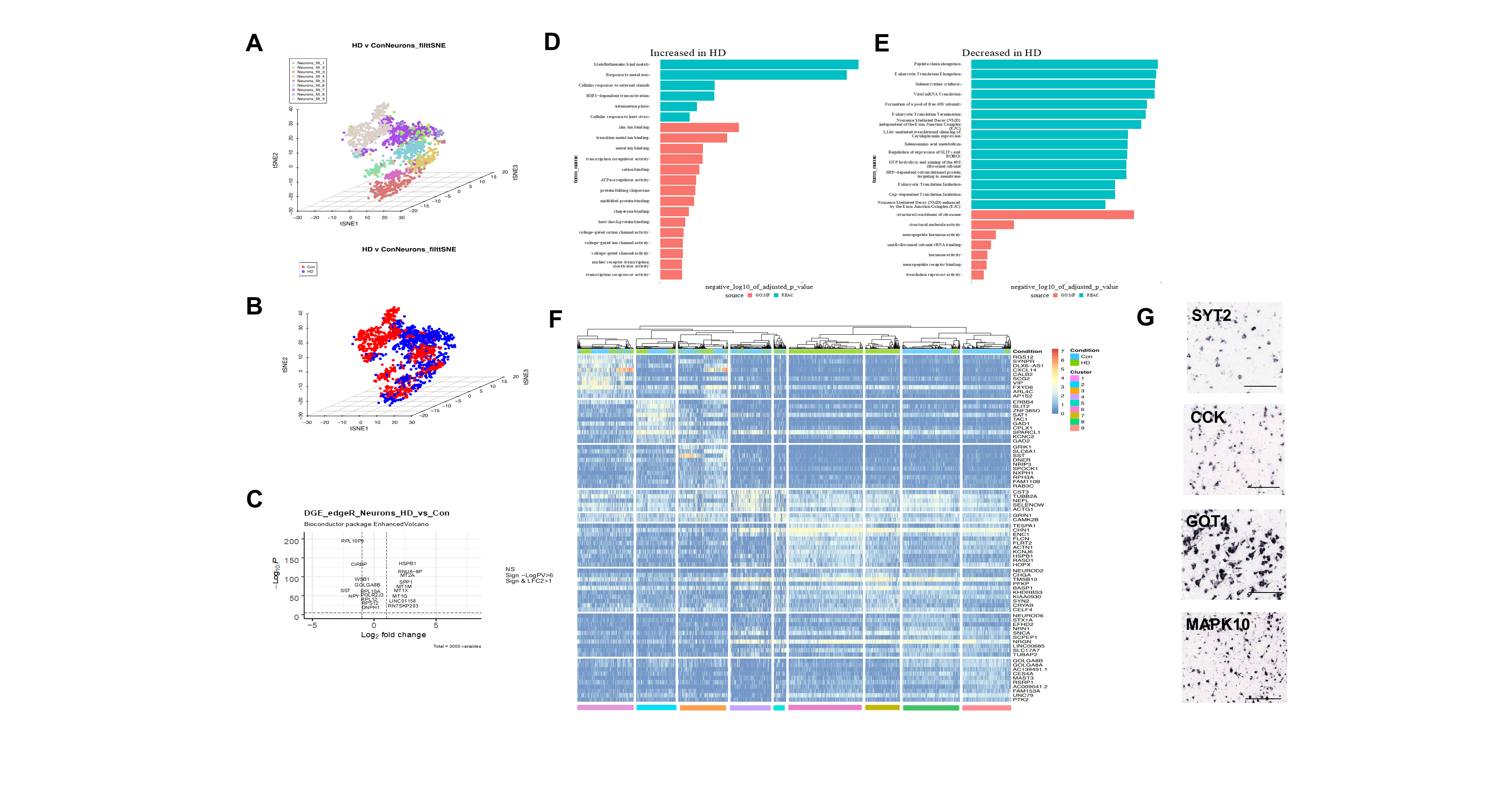

Supplement: Supplementary file 3 — Additional file 3. Differential gene expression patterns in neurons. A) tSNE plot showing 9 different neuronal clusters. B) Here we divided up nuclei in the tSNE plot into HD (blue) and control (red). Some of the clusters appear relatively homogeneous with respect to condition, while others appear more mixed. C) Differential gene expression as a volcano plot, showing some of the highly differentially expressed genes. D) GO terms and Reactome pathway enrichment analysis of genes significantly increased in HD over all neurons. E) GO terms and Reactome pathway enrichment analysis of genes significantly decreased in HD over all neurons. The source of the GO term is color coded. P value of enrichment is represented by the length of the bar. F) Gene expression heat map of cluster markers showing nuclei (Columns) and specific genes (Rows). Condition (Con versus HD) and neuronal clusters are color-coded on the top and bottom, respectively. Cluster-specific gene markers were identified using Wilcoxon signed rank test comparing gene ranks in the cluster with the highest mean expression against all others. p-values were adjusted using the “Holm” method. G) Examples of in situ hybridization of 4 of the neuronal genes (© 2010 Allen Institute for Brain Science. Allen Human Brain Atlas. Available from: human.brain-map.org). Scale bars: GOT1 100µm, others 200µm. [file 40478_2020_880_MOESM3_ESM.tiff]

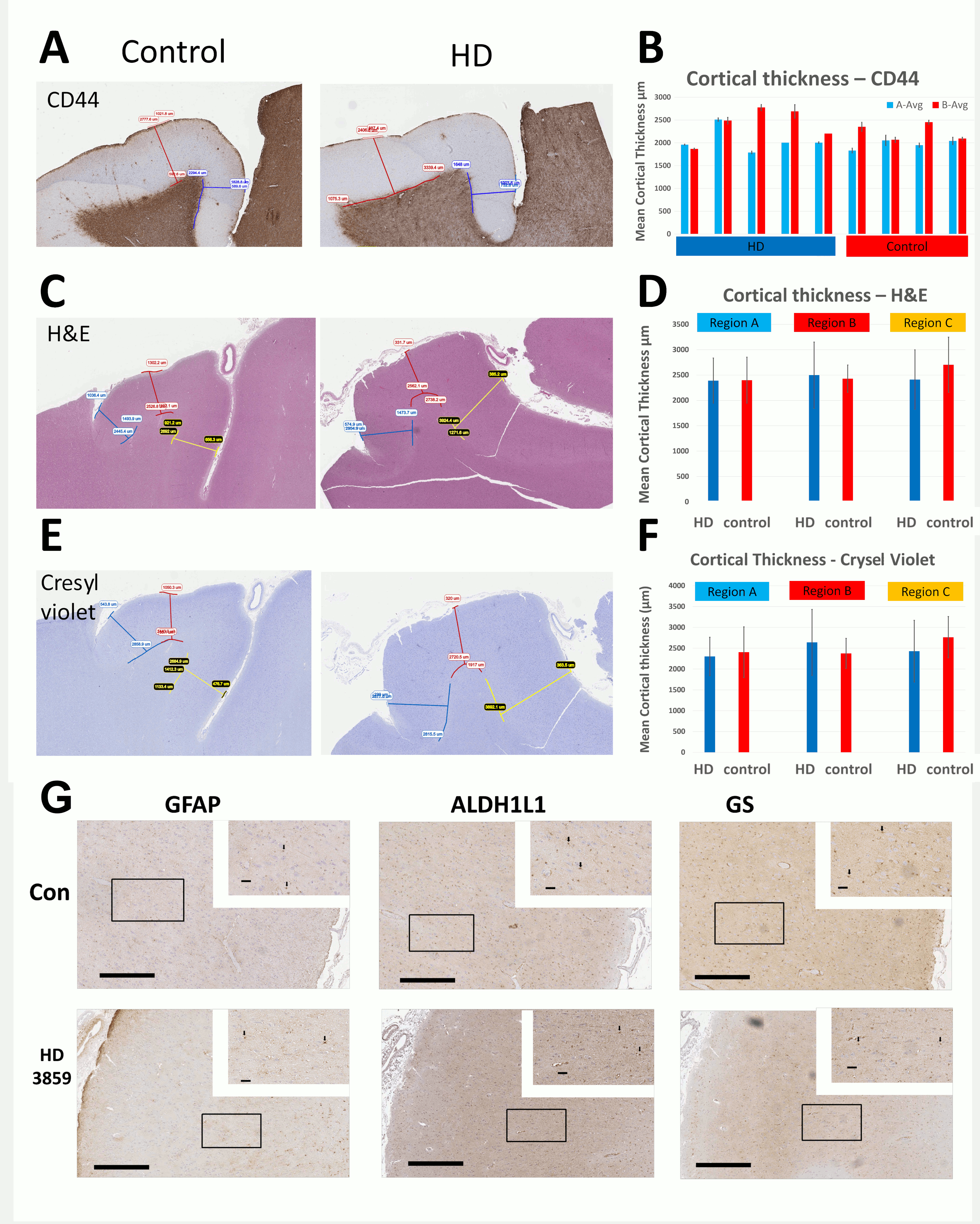

Supplement: Supplementary file 6 — Additional file 6. Cortical thickness of the cingulate in HD. Representative images of cortical thickness measurements performed in sections stained for CD44 (A), Hematoxylin and Eosin (H&E) (C), and Cresyl violet (E). Bar graphs showing average cortical thickness in individual cases in in the CD44 immunostain (B), with two regions quantified highlighted in blue and red. Bar graphs showing average cortical thickness of control and HD sections stained for H&E (D) and Cresyl violet (F). The regions quantified in the cingulate cortex are color-coded in the images, which is reflected in the bar graphs. No significant differences were identified between control and HD using unpaired t-tests. N =4 control and 5 HD for CD44 immunostain, 6-9 HD and 6-8 control for H&E, and 5-8 HD and 6-7 control Cresyl violet. G) Immunohistochemical staining for GFAP, Glutamine Synthetase (GS), and ALDH1L1 of a representative control and the Juvenile Huntington (T3859). Images are shown at 5X, and insets at 20X. Scale bars: 500μm, inset scale bar: 50μm. [file 40478_2020_880_MOESM6_ESM.tiff]

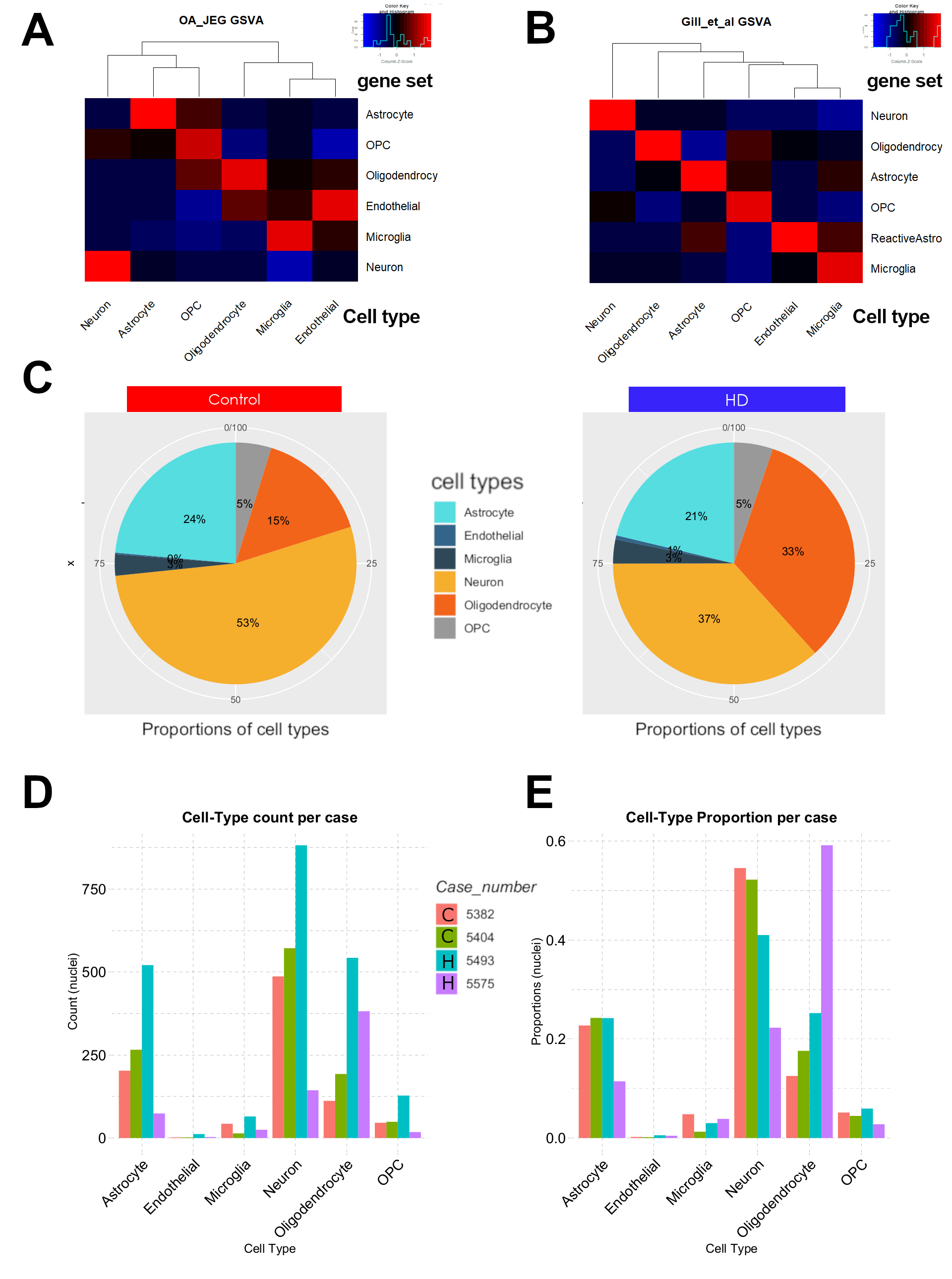

Supplement: Supplementary file 7 — Additional file 7. Gene set variation analysis (GSVA) of the average normalized expression of all nuclei in one cell-class/type. Cell-type specific gene sets derived from the literature (A OA and JEG) and Gill et al.53 (B) are shown in the rows. Cell-types are shown in columns. The z-scaled enrichment scores of the cell-type averages are shown in the heat maps (A-B). The proportions of cell-types in Control (Right) and HD (Left) nuclei. Percentages per cell-type are shown in the pie chart (C). Bar-plots of count of nuclei per cell-type per case (D). Barplots of the proportions of cell-type per case (C=Control, H=HD) (E) [file 40478_2020_880_MOESM7_ESM.tiff]

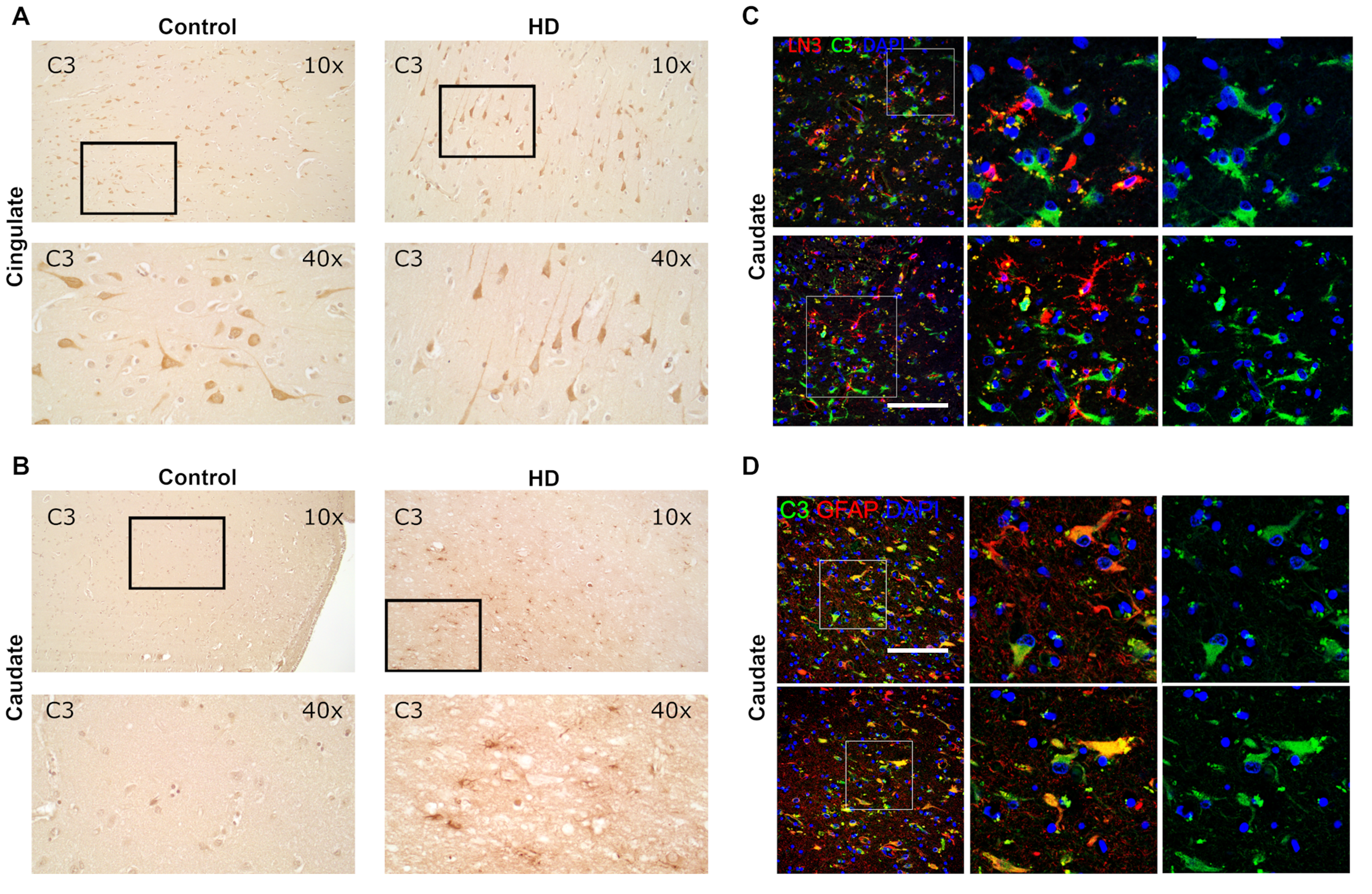

Supplement: Supplementary file 10 — Additional file 10. Complement factor 3 (C3) immunostaining in the HD caudate and cingulate. A-B) Micrographs of immunostaining for C3 in the cingulate cortex (A) and caudate nucleus (B) of control and HD grade III/IV taken at 10X (100X total magnification). The boxed areas are shown at 40X in the lower panels (400X total magnification). C-D) Dual immunostaining for C3 (green) and GFAP (red -C) or LN3 (red – D) in the caudate nucleus of a representative HD case (C). Nuclei stained with DAPI are shown in blue. Scale bars indicate ####. A total of 3-4 cases per group were examined. [file 40478_2020_880_MOESM10_ESM.tiff]

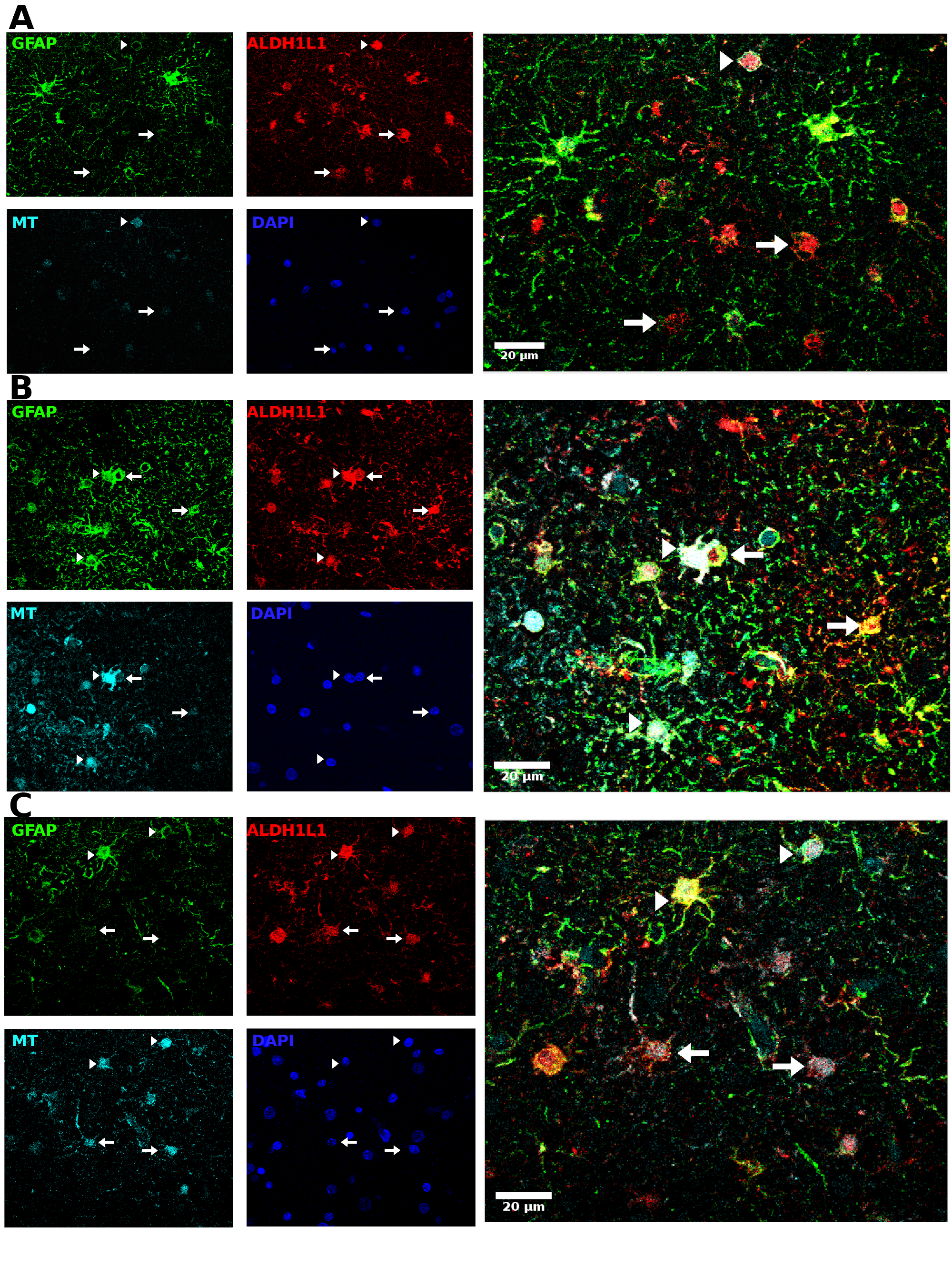

Supplement: Supplementary file 13 — Additional file 13. Validation of astrocytic sub-clusters. A) Astrocytes in a control cingulate cortex. Arrows indicate astrocytes that are ALDH1L1+/MT-/GFAP- (example of Astrocyte Clusters 3 or 4). The arrowhead indicates an astrocyte that is ALDH1L1+/MT+/GFAP-weak (example of Astrocyte Cluster 1). The two large reactive GFAP+/MT- astrocytes (not indicated by arrows) are examples of cluster 6. A merged panel is displayed on the right. B) Astrocytes in an HD cingulate cortex. Arrows indicate astrocytes that are ALDH1L1+/MT-weak/GFAP+ (example of Astrocyte Cluster 5). Arrowheads indicate astrocytes that are ALDH1L1+/MT+/GFAP+ (example of Astrocyte Cluster 2). C) Astrocytes in an HD cingulate cortex. Arrows indicate astrocytes that are ALDH1L1+/MT+/GFAP- or weak (example of Astrocyte Cluster 1). Arrowheads indicate astrocytes that are ALDH1L1+/MT+/GFAP+ (example of Astrocyte Cluster 2). GFAP (green), ALDH1L1 (red), MT (cyan), DAPI (white). Confocal microscopy in all panels; single optical planes are shown. Scale bar = 20µm. [file 40478_2020_880_MOESM13_ESM.tiff]

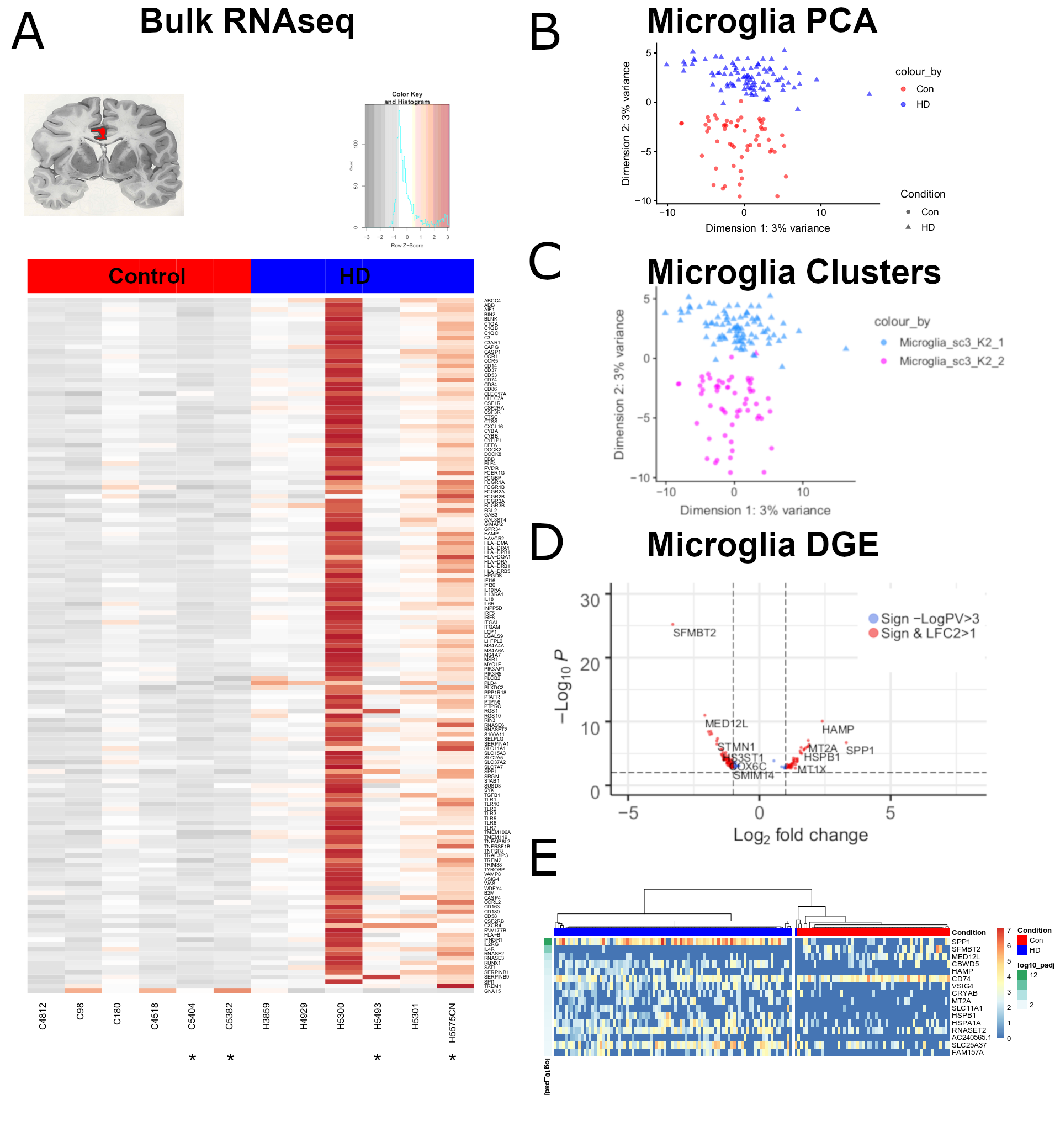

Supplement: Supplementary file 14 — Additional file 14. Microglial gene expression alterations in the cingulate cortex. A) Differential gene expression heatmap showing a subset of significantly differentially expressed microglial genes in control and HD cingulate cortex – cingulate cortex shown in red in the top right pictogram (Microglial gene list was adapted from Patir et al [40]). B) Principle component analysis plot of microglia nuclei. Control nuclei are shown in red, HD in blue. C) Clustering of control (circles) and HD (triangles) nuclei shows they cluster separately. Colors denote clusters as determined by SC3 consensus clustering (K=2). D) Differential genes expression of between control and HD microglial nuclei displayed as a volcano plot with significance set at p<0.05 – genes with –log10 p value (LogPV) of >3 are shown in blue, and those with -logPV of >3 and log2 fold change >2 are shown in red. Analysis was performed in EdgeR using the likelihood ratio test. E) Differential genes expression heatmap between control (red bar) and HD (blue bar) nuclei showing significantly differentially expressed genes (-log10 p value scale shown on the left). Significance was determined using Kruskal-Wallis test (p<0.05). [file 40478_2020_880_MOESM14_ESM.tiff]

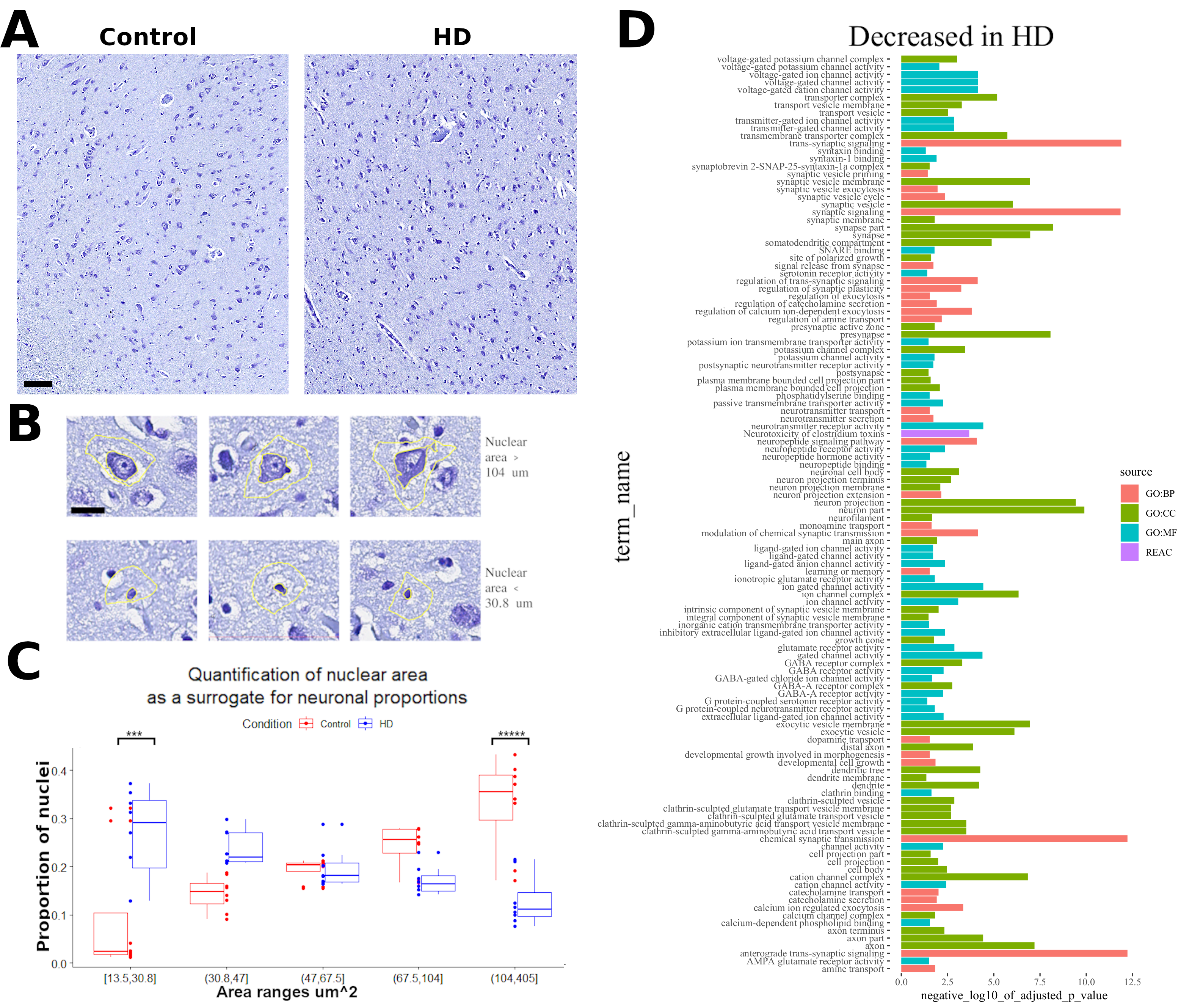

Supplement: Supplementary file 15 — Additional file 15. Neuronal loss and dysfunction in the HD cingulate cortex. A) Representative images of crystal violet stained cingulate cortex sections from a control case and a grade 4 HD. The subcortical white matter is shown in the lower right corner. Note the relative abundance of the small “glial” nuclei in the HD cortex. Scale bar = 100um. B) Representative images of cells with large nuclear area (5th quantile >104 um2) in the upper row and cells with small nuclear area (1st quantile <30.8 um2) in the lower row. These areas correspond to neurons and glia, respectively. C) Quantification of the relative proportions of nuclei quantified (y-axis) within different area ranges (x-axis). Boxplots are shown in addition to points representing individual cases. Clue indicates HD and red controls. N=9 for control N=8 for HD. ***: p value < 0.001, *****: p value < 0.000001. D) GO term and Reactome pathway enrichment analysis of genes significantly downregulated in HD in the bulk RNAseq analysis of the cingulate cortex. All of the pathways shown are significantly enriched after Benjamini-Hochberg False discovery rate correction (<0.05). The source of the GO term is color coded. P value of enrichment is represented by the length of the bar per gene ontology. [file 40478_2020_880_MOESM15_ESM.tiff]
