# Supplementary material for: Single-nucleus RNA-seq identifies Huntington disease astrocyte states
Source: Acta Neuropathol Commun. 2020 Feb 18;8:19. doi: 10.1186/s40478-020-0880-6 (PMC7029580; doi:10.1186/s40478-020-0880-6)
Supplement: Supplementary file 4 — Additional file 4. Supplementary Data. (1) Raw counts and RPKM counts of Bulk RNAseq data. (2)Results of differential gene expression analysis of each astrocytic cluster against all other clusters. [file 40478_2020_880_MOESM4_ESM.zip › Supplementary Data.docx]

# **Supplementary Data**

## **Supplementary Methods**

### **Quantification of cortical thickness and neuronal densities and statistical analysis**

To quantify cortical thickness, the boundary between the grey and white matter was first identified by a neuropathologist (OAD, JEG) on Hematoxylin and Eosin histochemical stain, Cresyl violet histochemical stain, and CD44 immunohistochemical stain. Whole slides were scanned and the images were analyzed in the Aperio™ eSlideManager. A set of lines orthogonal to the pial surface were drawn from the surface to the white matter, and the lengths of these lines were measured. The cingulate cortex was divided into three adjacent regions; abutting the corpus callosum, the convexity of the cingulate gyrus facing the contralateral gyrus, and the sub-frontal surface of the dorsal side of the cingulate gyrus. Three to five measurements were taken per region. Areas with staining artifact or tissue folding were excluded. The average cortical thickness for each region was calculated per case per stain. The number of cases examined was as follows: 4 control and 5 HD for CD44 immunostain, 6-9 HD and 6-8 control for H&E, and 5-8 HD and 6-7 control Cresyl violet. Unpaired t-test with unequal variance was performed to test significance.

For quantification of nuclear densities in whole slide images of Cresyl violet stained sections scanned at 20X, we used a semi-automated method using Qupath v0.2[1]. We employed a multi-step approach to first count cells in a ROI, agglomerate (bin) cells counts based on cell area, divide by total count, and finally express these counts as proportions of cells in given area range to the total count. In brief, images were loaded under the “bright field (other)” setting. The staining vector was estimated for each image from a representative region of interest (ROI), excluding unrecognized colors. The Cresyl violet signal was largely identified in the range of Hematoxylin. Next, a script to automate watershed cell detection was used with the following parameters based on “Optical density sum”: Background radius 8μm, Sigma 1.5μm, maximum area 600μm^2^, Threshold 0.2, smooth cell boundaries = True, and separate by shape = False. The results were collected for 3-6 ROI per image. Areas with tissue folds, staining artifact, or blood vessels were excluded. One image from each patient was used. The results were loaded in R v3.6. An empirical threshold of the mean nuclear hematoxylin intensity and sum nuclear hematoxylin intensity was determined to exclude cells/events that represented tangential cuts through cells or background noise. The mean nuclear sum and mean hematoxylin intensity were first normalized. The threshold was determined based on inspecting values from 4 images (and validated in the remaining images). All events were first filtered by the threshold determined for the sum of the hematoxylin intensity, followed by the threshold determined for the mean nuclear hematoxylin intensity. Next, the events of nuclear area (Hematoxylin) were tallied and grouped based on 5 quantiles based on the range of nuclear areas. The counts per case were normalized by the total event count per case to get the relative proportions of nuclei in each of the 5 quantiles of area ranges and analyzed by a two-way ANOVA with an unbalanced design (n= 9 for control and 8 for HD), and ANOVA type set at “III”. The test was performed in R, with the counts per area range as the dependent variable, and the area ranges and Conditions as explanatory variables, with an interaction term between area ranges and Condition. Tuckey test for posthoc testing was done and statistical results were only reported for results of the interaction comparisons.

For quantification of GFAP + cell densities in GFAP/HTT double stained or GFAP single immunostained slides, we counted cells with an area exceeding an empirically-determined threshold, which corresponds to astrocytes with increased GFAP-immunopositive cell area- associated with reactive astrocytosis. The following script was used in QuPath v0.2: runPlugin('qupath.imagej.detect.cells.WatershedCellDetection', ''). For DAB stained slides, the following empirically determined setting were used: runPlugin('qupath.imagej.detect.cells.WatershedCellDetection', '{"detectionImageBrightfield": "Optical density sum", "requestedPixelSizeMicrons": 0.5, "backgroundRadiusMicrons": 10.0, "medianRadiusMicrons": 1.5, "sigmaMicrons": 1.5, "minAreaMicrons": 40.0, "maxAreaMicrons": 600.0, "threshold": 0.4, "maxBackground": 2.0, "watershedPostProcess": false, "excludeDAB": false, "cellExpansionMicrons": 7.67, "includeNuclei": true, "smoothBoundaries": true, "makeMeasurements": true}'). The counts and areas of regions of interests were entered into a Table into R. Cell densities were calculated. Mann-Whitney U test (one-sided) was used to calculate statistical significance.

**Quantification of GFAP and MT optical density**

Quantification of the levels of GFAP and MT immunoreactivity was performed on the images obtained with confocal microscope from triple (GFAP/MT/ALDH1L1) stained sections. Images (merged from the z-stacks of adjacent 6 optical planes [1024 × 1024 pixel resolution, observed area 606 × 606 μm of a 40x field] captured at a z plane distance of 0.4 μm from each other) were transferred into Image J (public domain), grayscaled for each channel, and optical density (OD) was determined in a circle area (diameter 30 μm) centered at the cell nuclei. Only astrocytes with clearly outlined nuclei profiles (DAPI staining) were taken into consideration. At least 5 images from randomly selected gray matter areas from 3 cases of HD and 3 cases of control cingulate cortex were used for quantification. A total of 79 cells (HD) and 49 (control) cells were analyzed. An unpaired two-tailed t-test was used for statistical comparison.

## **Supplementary Results**

### **Neuronal loss and transcriptional alterations in the HD cingulate cortex**

Here we present neuronal data, since neuronal pathology in HD has been the major object of most studies. First, to determine the extent of neurodegeneration in our samples of cingulate cortex, we examined the cortical thickness in different histochemical stains of control and HD tissue. The results showed no significant difference in cortical thickness of the anterior cingulate gyrus between control and HD cases as quantified with a CD44 immunohistochemical stain (**Supplementary Figure 1A-B**), an H&E histochemical stain (**Supplementary Figure 1A, 1C-D**), and a Cresyl violet histochemical stain (**Supplementary Figure 1C, 1E-F**). Next, we quantified the relative proportions of nuclei of different sizes in Cresyl Violet stained sections of the cingulate cortex (**Supplementary Figure 4A)**. We categorized the nuclear areas into 5 quantiles. Larger nuclear areas corresponded to pyramidal neurons, whereas smaller areas were either glial or small neuronal nuclei (**Supplementary Figure 4B**). We next conducted a two-way ANOVA analysis of the proportion of nuclei in each area bin between control and HD. Although there were no overall significant effects of the Condition (F ratio: 1.2036 and p value 0.317) or Area range (F ratio 0 and p value 1), there is significant crossover interaction (F ratio of 17.635 and p value 4.72e-10). The proportion of nuclei in an area range (bin) is the opposite, depending on the condition (**Supplementary Figure 4C**). More specifically, the proportion of nuclei within a 13.5-30.8µm^2^ range were increased in HD (Adjusted p value 1.7 e-4), while the proportion of nuclei with areas greater than 104 µm^2^ were decreased in HD compared to control (Adjusted p value 7.6 e-6 – **Supplementary Figure 4C**). The increased proportion of small nuclei could reflect a shrinkage of larger cells or a greater proportion of small, glial cells, or both. We have independent evidence that the proportion of cells of the oligodendrocyte lineage is increased, and is accompanied by a shift to less mature oligodendrocytes (manuscript in preparation).

We first analyzed our bulk RNASeq data, examining the gene ontology terms and Reactome pathways enriched in genes downregulated in HD. The majority of terms relate to neuronal identity (synapse, neuron part, neuron projection, axon part, dendrite tree, and growth cone, for eg.) or neuronal function (GABA receptor complex, regulation of catecholamine secretion, dopamine transport, AMPA glutamate receptor activity, and neuropeptide receptor activity). These GO terms are presented in **Supplementary Figure 4D.** Together, these data suggest that neuronal numbers and function are reduced in these HD cingulate cortex samples.

We then analyzed snRNASeq findings. A total of 1866 neuronal nuclei passed quality control. When neuronal nuclei were placed in a tSNE plot, we distinguished 9 different neuronal clusters (**Supplementary Figure 5A**). HD clusters and control clusters were largely separated, with some overlap (**Supplementary Figure 5B**). A differential gene expression analysis (volcano plot) revealed a number of up- and down-regulated genes in comparing HD neurons with control neurons. The former included MT and heat shock protein genes, as we found in astrocytes, the latter included somatostatin and NPY (**Supplementary Figure 5C**). Analysis of enriched gene ontology terms and Reactome pathways showed the top gene ontology molecular function terms of genes increased in HD included terms related to MTs, metal binding, heat-shock factor 1 (HSF-1) dependent transactivation, misfolded protein binding, molecular chaperones, and, interestingly, voltage gated ion channel activity (**Supplementary Figure 5D**). Gene ontology terms of genes decreased in HD included those related to translation initiation and elongation, selenoamino acid metabolism, regulation of SLITs and ROBOs, structural constituents of ribosomes, and neuropeptide receptor binding (**Supplementary Figure 5E**). A list of differentially expressed genes in HD versus control neurons as well as the full results of GO term enrichment analysis are provided in **Supplementary Table 6B-C**. A list of the top neuronal cluster markers is provided in **Supplementary Table 7.**

Differential gene expression of the neuronal clusters was displayed in a heat map (**Supplemental Figure 5F**). Clusters 1, 2, and 3 show high expression of genes characteristic of inhibitory interneurons (for example, *VIP*, *GAD1*, *CALB2*, *SST*). Clusters 4-9 are relatively much lower in these transcripts, and higher in a set of other transcripts (for eg. *SLC17A7*, *NGRN*, *CHN1*, *SNCA*, *NEFL*, *GRIN1*, *TMSB10*, *BASP1*), associated with excitatory neurons. However, an inspection of the heat map does show gene expression differences that vary among these clusters, suggesting significant variation in populations of excitatory neurons. The clusters that correspond to inhibitory neurons contain mixtures of cells from control and HD brains, while clusters from excitatory neurons are divided up much more clearly between control and HD brains. This suggests that changes in excitatory neuron gene expression is more affected by HD than inhibitory neuron gene expression. We validated that a number of the transcripts that we found associated with the neuronal clusters were indeed expressed by neurons by referring to *in situ* hybridizations from the Allen Brain Atlas (**Supplementary Figure 5G**).

### **A subset of Microglial genes is captured by single nuclei RNAseq in the cingulate HD cortex**

Because of the inflammatory gene expression in HD and because both astrocytes and microglia contribute to inflammation, we are presenting findings on microglial gene expression. Investigation of microglial gene expression in bulk RNAseq data based on a list of specific microglial genes from Patir, et al.[2] showed a significant increase in multiple immune genes (**Supplementary Figure 6A**). Multiple immune pathways were enriched in HD including IL-4, IL-10, and IL-13 signaling (**Figure 1D**). There is significant heterogeneity between HD cases; with the signature being largely driven by two HD cases (5300 and 5575). The genes increased in HD included *TLR*, *HLA*, Fc Gamma receptors, and complement factor genes. We investigated the individual transcriptional profiles from snRNAseq of HD cases 5575 and 5493 and controls 5382 and 5404. We identified 147 nuclei as microglia (3.7% of total nuclei, **Supplementary Figure S6B**). Control and HD nuclei clustered separately (**Supplementary Figure S6C**). Differential gene expression analysis showed 148 genes to be differentially expressed at a false discovery rate of 0.25 (**Supplementary Figure S6D-E**). From the differentially expressed genes, seven genes were found to be also upregulated in the bulk RNAseq data (*SPP1*, *CD163*, *SLC11A1*, *VSIG4*, *HLA-DRB5*, *FCGR3A*, *MS4A6A*). Three genes were downregulated compared with the bulk RNAseq (*CSF3R*, *CSF2RA*, and *TREM2*). Note this data reflect gene expression in nuclei from two HD cases and two controls, as opposed to n=6 per group in bulk RNAseq. The results indicate that with our approach, detecting microglial gene alterations at the single nucleus level might not be as sensitive as bulk RNA sequencing. The genes used in the heatmap of **Supplementary Figure 6** are provided in **Supplementary Table 7**.

A limitation we note in our study is that we detected only a small number of microglial nuclei. The paucity of microglial nuclei and the sparsity of nuclear reads likely explains why we did not detect many differentially expressed microglial genes. In addition, only one of the two HD donor brain samples showed large upregulation of microglia reads in the bulk RNAseq analysis. Thus, our approach may not be powered to illuminate transcriptional alterations of microglia at the single nucleus level. That being said, we still found alterations of the immune system using the bulk RNAseq data.

### **HD modifier genes expression in neurons and astrocytes**

We investigated the expression of HD modifier genes in neurons and astrocytes (Gem-HD consortium, 2015). We note that while there were no detected changes in the expression of HTT between control and HD neurons, HD astrocytes overall expressed lower levels of HTT (log fold change = -0.439, FDR=0.0189). Next, we turned our attention to neurons. We detected no significant changes in the expression of HD modifier genes *FAN1*, *RRM2B*, or *UBR5* in HD neurons versus control (**Supplementary table 6A**). Conversely, the expression of *MLH1* was significantly reduced in HD neurons compared with controls (log fold change = -0.449, FDR = 2.99 E-5; **Supplementary Table 6A**). The expression of *MLH1* was mostly noted in the control neuronal clusters 7 and 8 (excitatory neurons – data not shown). The picture is different in astrocytes (**Supplementary Tables 2C).** We found, the expression of both *MTMR10* and *UBR5* were reduced in HD astrocytes compared to controls (*MTMR10* log fold change = -0.409, FDR = 0.014, UBR5 log fold change -0.673, FDR = 3.18 E-5). The expression of *FAN1* or *RRM2B* showed no significant difference between HD and control astrocytes. Interestingly, *HTT*, *UBR5*, and *MLH1* were not differentially expressed between HD and controls at our threshold in bulk RNAseq (absolute log fold change levels > 0.5). In contrast, MTMR10 was significantly increased (log fold change = 0.89 and FDR=0.015 - **Supplementary Table 5**). In conclusion, the data reveals significant difference between HD astrocytes and neurons at the single nucleus level; HD astrocytes expressed lower levels of *HTT* and the disease modifiers *UBR5* and *MTMR10*. Conversely, HD neurons did not show significant changes in *HTT*, but showed overall lower expression of the disease modifier *MLH1*. These changes were not reflected in differential gene expression changes in bulk RNAseq, which is a representation of both gene expression per cell type as well as cell type proportions. This may underlie the differences we see in *MTMR10, MLH1, UBR5*, and *HTT* in snRNAseq versus bulk RNAseq.

### **Cell type-specific markers derived from nuclear transcripts**

We provided a list of astrocyte sub-cluster markers (“**Consensus clustering using SC3 for sub-clustering”, above,** and **Table 2**). We also derived sets of cell type cluster markers (**Supplementary Table 1C**). For this, we used the cell-type gene lists from neurons, astrocytes, microglia, oligodendrocytes, OPCs, and endothelial cells. Note that there are many overlaps between this list and the original, literature-based, list we used for the Cell Classifier (**Supplementary Table 1B**), but some genes appear in one but not the other. These differences may result from different levels of transcripts in the nucleus vs. cytoplasm and nucleus combined (for single cell RNASeq).

**Supplementary Figure Legends**

### **Supplementary Figure 1:**

**Cortical thickness of the cingulate in HD**. Representative images of cortical thickness measurements performed in sections stained for CD44 (**A**), Hematoxylin and Eosin (H&E) (**C**), and Cresyl violet (**E**). Bar graphs showing average cortical thickness in individual cases in in the CD44 immunostain (**B**), with two regions quantified highlighted in blue and red. Bar graphs showing average cortical thickness of control and HD sections stained for H&E (**D**) and Cresyl violet (**F**). The regions quantified in the cingulate cortex are color-coded in the images, which is reflected in the bar graphs. No significant differences were identified between control and HD using unpaired t-tests. N =4 control and 5 HD for CD44 immunostain, 6-9 HD and 6-8 control for H&E, and 5-8 HD and 6-7 control Cresyl violet. **G**) Immunohistochemical staining for GFAP, Glutamine Synthetase (GS), and ALDH1L1 of a representative control and the Juvenile Huntington (T3859). Images are shown at 5X, and insets at 20X. Scale bars: 500μm, inset scale bar: 50μm.

### **Supplementary Figure 2:**

**Related to Figure 2. Gene set variation analysis (GSVA) of the average normalized expression of all nuclei in one cell-class/type.** Cell-type specific gene sets derived from the literature (**A** OA and JEG) and Gill et al.^53^ (**B**) are shown in the rows. Cell-types are shown in columns. The z-scaled enrichment scores of the cell-type averages are shown in the heat maps (**A-B**). The proportions of cell-types in Control (Right) and HD (Left) nuclei. Percentages per cell-type are shown in the pie chart (**C**). Bar-plots of count of nuclei per cell-type per case (**D**). Barplots of the proportions of cell-type per case (C=Control, H=HD) (**E**)

### **Supplementary Figure 3:**

**Complement factor 3 (C3) immunostaining in the HD caudate and cingulate**. **A-B**) Micrographs of immunostaining for C3 in the cingulate cortex (**A**) and caudate nucleus (**B**) of control and HD grade III/IV taken at 10X (100X total magnification). The boxed areas are shown at 40X in the lower panels (400X total magnification). **C-D**) Dual immunostaining for C3 (green) and GFAP (red -**C**) or LN3 (red – **D**) in the caudate nucleus of a representative HD case (**C**). Nuclei stained with DAPI are shown in blue. Scale bars indicate ####. A total of 3-4 cases per group were examined.

### **Supplementary Figure 4:**

**Neuronal loss and dysfunction in the HD cingulate cortex**. **A**) Representative images of crystal violet stained cingulate cortex sections from a control case and a grade 4 HD. The subcortical white matter is shown in the lower right corner. Note the relative abundance of the small “glial” nuclei in the HD cortex. Scale bar = 100um. **B**) Representative images of cells with large nuclear area (5^th^ quantile >104 um^2^) in the upper row and cells with small nuclear area (1^st^ quantile <30.8 um^2^) in the lower row. These areas correspond to neurons and glia, respectively. **C**) Quantification of the relative proportions of nuclei quantified (y-axis) within different area ranges (x-axis). Boxplots are shown in addition to points representing individual cases. Clue indicates HD and red controls. N=9 for control N=8 for HD. ***: p value < 0.001, *****: p value < 0.000001. **D**) GO term and Reactome pathway enrichment analysis of genes significantly downregulated in HD in the bulk RNAseq analysis of the cingulate cortex. All of the pathways shown are significantly enriched after Benjamini-Hochberg False discovery rate correction (<0.05). The source of the GO term is color coded. P value of enrichment is represented by the length of the bar per gene ontology.

### **Supplementary Figure 5:**

**Differential gene expression patterns in neurons. A)** tSNE plot showing 9 different neuronal clusters. **B)** Here we divided up nuclei in the tSNE plot into HD (blue) and control (red). Some of the clusters appear relatively homogeneous with respect to condition, while others appear more mixed. **C)** Differential gene expression as a volcano plot, showing some of the highly differentially expressed genes. **D)** GO terms and Reactome pathway enrichment analysis of genes significantly increased in HD over all neurons. **E)** GO terms and Reactome pathway enrichment analysis of genes significantly decreased in HD over all neurons. The source of the GO term is color coded. P value of enrichment is represented by the length of the bar. **F)** Gene expression heat map of cluster markers showing nuclei (Columns) and specific genes (Rows). Condition (Con versus HD) and neuronal clusters are color-coded on the top and bottom, respectively. Cluster-specific gene markers were identified using Wilcoxon signed rank test comparing gene ranks in the cluster with the highest mean expression against all others. p-values were adjusted using the “Holm” method. **G)** Examples of *in situ* hybridization of 4 of the neuronal genes (© 2010 Allen Institute for Brain Science. Allen Human Brain Atlas. Available from: [human.brain-map.org](http://human.brain-map.org/)). Scale bars: GOT1 100μm, others 200μm.

### **Supplementary Figure 6:**

**Microglial gene expression alterations in the cingulate cortex**. **A**) Differential gene expression heatmap showing a subset of significantly differentially expressed microglial genes in control and HD cingulate cortex – cingulate cortex shown in red in the top right pictogram (Microglial gene list was adapted from Patir et al [40]). **B**) Principle component analysis plot of microglia nuclei. Control nuclei are shown in red, HD in blue. **C**) Clustering of control (circles) and HD (triangles) nuclei shows they cluster separately. Colors denote clusters as determined by SC3 consensus clustering (K=2). **D**) Differential genes expression of between control and HD microglial nuclei displayed as a volcano plot with significance set at p<0.05 – genes with –log10 p value (LogPV) of >3 are shown in blue, and those with -logPV of >3 and log2 fold change >2 are shown in red. Analysis was performed in EdgeR using the likelihood ratio test. **E**) Differential genes expression heatmap between control (red bar) and HD (blue bar) nuclei showing significantly differentially expressed genes (-log10 p value scale shown on the left). Significance was determined using Kruskal-Wallis test (p<0.05).

### **Supplementary Figure 7:**

**Outline of the snRNAseq analysis pipeline**. Briefly, filtered raw un-clustered data is pre-clustered using a shared nearest neighbor algorithm. The pre-clusters are classified into cell classes/lineages using gene set enrichment analysis for specific lineage genes and examining GO terms of the top pre-clusters markers. Next, mixed pre-clusters are identified and the cells in these pre-clustered are re-classified based on the cell-specific lineage-scores (Cell classifier tool). Next, clusters of the same lineage are agglomerated and cell-classes/lineages are analyzed in isolation from the remaining cell classes using SC3 consensus clustering into sub-clusters (Astrocyte sub-clusters are shown as an example). These sub-clusters are used for downstream analysis.

**Supplementary Figure 8:**

**Validation of astrocytic sub-clusters**. **A**) Astrocytes in a control cingulate cortex. Arrows indicate astrocytes that are ALDH1L1+/MT-/GFAP- (example of Astrocyte Clusters 3 or 4). The arrowhead indicates an astrocyte that is ALDH1L1+/MT+/GFAP-weak (example of Astrocyte Cluster 1). The two large reactive GFAP+/MT- astrocytes (not indicated by arrows) are examples of cluster 6. A merged panel is displayed on the right. **B**) Astrocytes in an HD cingulate cortex. Arrows indicate astrocytes that are ALDH1L1+/MT-weak/GFAP+ (example of Astrocyte Cluster 5). Arrowheads indicate astrocytes that are ALDH1L1+/MT+/GFAP+ (example of Astrocyte Cluster 2). **C**) Astrocytes in an HD cingulate cortex. Arrows indicate astrocytes that are ALDH1L1+/MT+/GFAP- or weak (example of Astrocyte Cluster 1). Arrowheads indicate astrocytes that are ALDH1L1+/MT+/GFAP+ (example of Astrocyte Cluster 2). GFAP (green), ALDH1L1 (red), MT (cyan), DAPI (white). Confocal microscopy in all panels; single optical planes are shown. Scale bar = 20μm.

**Supplementary References**

1 Bankhead P, Loughrey MB, Fernández JA, Dombrowski Y, McArt DG, Dunne PD, McQuaid S, Gray RT, Murray LJ, Coleman HGet al (2017) QuPath: Open source software for digital pathology image analysis. Scientific Reports 7: Doi 10.1038/s41598-017-17204-5

2 Patir A, Shih B, McColl BW, Freeman TC (2019) A core transcriptional signature of human microglia: Derivation and utility in describing region‐dependent alterations associated with Alzheimer's disease. Glia 67: 1240-1253 Doi 10.1002/glia.23572
